# Karyotype of the Cricket, *Zucchiella atlantica*, with an Overview of the Chromosomes of the Subfamily Nemobiinae

**DOI:** 10.1673/031.007.6001

**Published:** 2007-12-03

**Authors:** C.B. Portugal, A. Mesa

**Affiliations:** ^1^ Universidad de La Rioja, CCT, Departmento Agricultura y Alimentación, Calle Madre de Dios, 51, 26006, Logroño, La Rioja, España; ^2^ Universidad Estadual Paulista, Institute Biociências, Departmento de Biologia, Av. 24-A, 1515, Bela Vista, Rio Claro, SP, Brazil, 13506-900

## Abstract

Few reports have been published on cytogenetics in crickets of the subfamily Nemobiinae. Within the Neotropical region the karyotypes of only two species are known, both of them belonging to the genus *Phoremia*. In the present paper, chromosomes of a third Neotropical species, *Zucchiella atlantica*Mello 1990 (Orthoptera: Trigonidiidae), have been studied and a cytological review of other species of that subfamily is presented. *Zucchiella atlantica* shows 2n ♂ = 22 + XO and 2n ♀ = 22 + XX which suggests an ancestral condition within the subfamily as the diploid number in all the species previously studied ranges from 2n ♂ = 7 to 2n ♀ = 21. In Orthoptera those species with high chromosome numbers tend to show reduction in their chromosomal numbers by means of centric fusions rather than to increase chromosomal numbers, due to difficulties in the availability of new centromeres. A structural polymorphism in one chromosome of pair 5 was observed as an intra-individual variation, suggesting differential activity of the genome from cell to cell.

## Introduction

The order Orthoptera includes the superfamily Grylloidea with several families. Trigonidiidae is one of its families and has been divided into two subfamilies: Trigonidiinae and Nemobiinae. The latter aggregates six tribes, three of which, Hemigryllini, Nemobiini and Pteronemobiini, are represented by several species in the Neotropical region.

The genus *Zucchiella* with its single species, *Z. atlantica*, was described by Mello (1990) and included by this author in the tribe Nemobiini due to the absence of a specialized glandular spur on male hind tibiae. *Z. atlantica* is a small wingless species, living preferentially in dense and shaded leaf litter in Atlantic rain forests, in the regions of Ubatuba and Caraguatatuba (São Paulo State, Brazil).

Several papers have been published about chromosomes of the subfamily Nemobiinae, most of them from Asia and North America. Of the 51 genera within in the Nemobiinae, only nine of them have been partially analyzed from the cytogenetical point of view. In the Neotropical region only two species of the genus *Phoremia* have been cytologically analyzed (Mesa and Ribas, 1996; Mesa et al., 1999).

The present paper deals with the study of mitotic and meiotic chromosomes of *Z. atlantica* as well as a revision of the karyological information from other species of its subfamily.

## Material and Methods

Specimens of *Z. atlantica* were collected at Ubatuba (São Paulo State, Brazil). Nymphs and adults were removed from the leaf litter of an abandoned cacao plantation. 37 males and 21 females from the same population in space and from different generations were cytologically analyzed.

Collecting data: “Fazenda Capricórnio”, Ubatuba, São Paulo, Brazil (23°23′21″S - 45°04′31″W), 26.8.1996, A. Mesa, P. García, C.F. Sperber, C. C. Ribas - 1 male. *Idem*, 06.10.1996, A. Mesa, P. Garcia - 1 male. *Idem*, 05.11.1996, A. Mesa, P. Garcia - 2 males. *Idem*, 05.9.1999, C. B. Portugal, A. R. Miyoshi - 2 males. *Idem*, 22.7.1999, A. Mesa, E. Zefa, C. B. Portugal, A. R. Miyoshi - 6 males and 3 females. *Idem*, 21.5.2000, C. B. Portugal, A. R. Miyoshi - 5 males. *Idem*, 21.11.2001, C. B. Portugal, A. R. Miyoshi - 6 females. *Idem*, 21.11.2001, C. B. Portugal, A. R. Miyoshi - 4 females. *Idem*, 09.12.2003, C. B. Portugal - 1 male. *Idem*, 22.January.2003, C. B. Portugal - 1 male and 1 female. *Idem*, 23.8.2003, C. B. Portugal, A. R. Miyoshi - 8 males and 5 females. *Idem*, 09.4.2004, C. B. Portugal, A. R. Miyoshi - 10 males and 2 females.

Male meiotic cells have been obtained from testes and the mitotic cells from cecum and ovary tissues of colchicine injected specimens (0.05% in insect physiologic solution). The material was fixed and preserved in Carnoy I (3:1, methanol and acetic acid) at -20°C for further analysis. Tissues were softened and the cells were dispersed in 45% acetic acid, centrifuged and fixed twice after discarding the supernatant medium. Slides were prepared as described by Henegariu et al. 2001, with modifications. Chromosome morphology was determined according to White (1973).

## Results and Discussion

**Figure 1.**
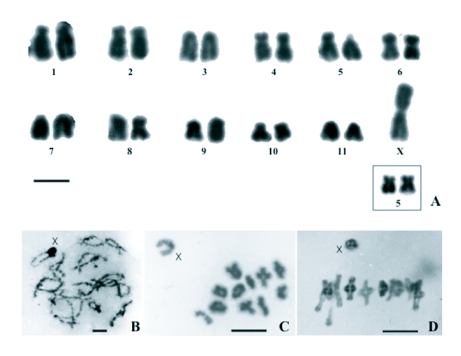
Chromosomes of *Zucchiella atlantica* Mello, 2n = 22 + XO (males) - 2n = 22 + XX (females). (A) Male karyotype, heteromorphic for the pair 5. Box showing the pair 5 in homomorphical condition. (B) Early diplotene. (C) Diakinesis. (D) Early first anaphase. Scale bar = 10 µm.

The karyotype of *Z. atlantica* is 2n ♂ = 22 + XO and 2n♀ = 22 + XX is shown in Figure 1A. Chromosomal morphology was determined as follows: the pairs 2, 4, 5, 6, 8 and 10 are submetacentric, the pairs 1, 3, 7, 9 and 11 are acrocentric and the sex chromosome is metacentric. The sexual chromosome is metacentric with equal arms, and is the largest of the complement. Pair 5 is heteromorphic under conventional staining of C-metaphase in both sexes, appearing as submetacentric-submetacentric (SM-SM) and as submetacentric-acrocentric (SM-A), as an intra-individual variation (see karyotype and attached box in Figure 1A). General aspects of male meiotic cells at diplotene (Figure 1B), diakinesis (Figure 1C) and early first anaphase (Figure 1D) are shown.

Among more than 50 genera placed in the subfamily Nemobiinae, 31 species belonging to nine genera have been cytologically studied. Many cases of synonymy or taxonomical non-concordance have been described by different authors. An updated taxonomy (Otte et al. (2001) of these species is compiled in Table 1, with available information about their karyotypes.

The sex-determining mechanism is XO (males)-XX (females) in every case studied in this subfamily, showing that the autosomes are involved in karyotypical evolution instead of the sexual chromosomes. This is expected because once sexual chromosomes cannot undergo centric fusions with autosomes to create neo-XY systems, XO/XX sex determination is preserved in the subfamily Nemobiinae as a whole.

**Table 1.**
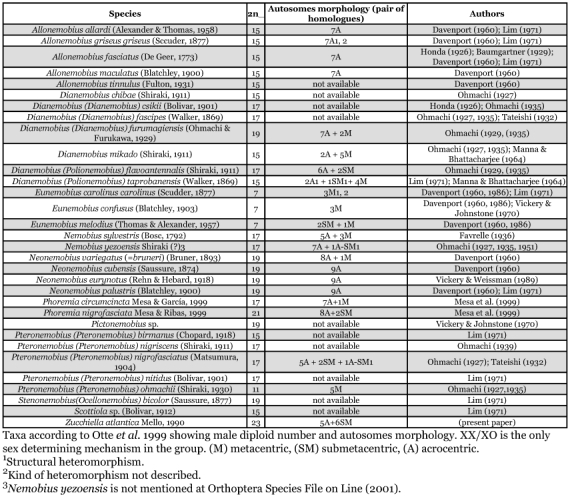
List of Nemobiinae crickets cytologically studied.

The male diploid number in 31 species of the subfamily Nemobiinae ranges from 2n = 7 to 2n = 21, with the following percentage: three species (9,7%) with 2n = 7, one species (3,2%) with 2n = 11, ten species (32,3%) with 2n = 15, nine species (29,0%) with 2n = 17, seven species (22,6%) with 2n = 19 and one species with 2n = 21 (3,2%). Such data demonstrate the high heterogeneity of chromosomal numbers in Nemobiinae, that, presumibly, impedes the establishment of a standard number in this group. *Z. atlantica* presents the highest chromosome number known within the Nemobiinae. It is known that trends in Orthoptera species are to reduce chromosome numbers, by means of centric and tandem fusions, rather than increasing the chromosomal number. The *Z. atlantica* karyotype probably represents an ancestral form in the group.

In meiotic prophase the sexual chromosome seems to have a large block of procentric heterochromatin, whereas some of the autosomal bivalents display terminal, and some distal, heterochromatin (Figure 1D). Male meiotic cells at diplotene show evidence of most bivalents having 2 or 3 chiasmata (Figure 1B) and it is not possible to determine exactly which autosome corresponds to each bivalent because of their similar and small sizes. At diakinesis and at early first anaphase (Figure 1C and 1D) the cells apparently show bivalents with only one chiasma. This is expected at meiosis in Orthoptera because the number of chiamata can vary as meiosis develops, and in this case it diminishes at final stages. Furthermore, as in these bivalents the chromosomes have terminal heterochromatin. Terminal achiasmatic associations are displayed at early diplotene which is dislocated at repulsion in late diplotene and so is not seen at the later stages.

Polymorphisms for structural rearrangements in Nemobiinae involving autosomes have been described for the following species: *Allonemobius g. griseus* (Davenport, 1960; Lim, 1971), *Dianemobius* (*Polionemobius*) *taprobanensis* (Lim, 1971; Manna & Bhattacharjee, 1964), *Eunemobius c. carolinus* (Davenport, 1960, 1986; Lim, 1971), *Nemobius yezoensis* (Ohmachi, 1927a; 1935), *Pteronemobius nigrofasciatus* (Ohmachi 1927a; Tateishi, 1932). For the Neotropical fauna Mesa et al. 1999 reported the occurrence of a heteromorphism involving the fifth pair of autosomes in *Phoremia nigrofasciata* (Table 1).

In *Z. atlantica* it has been observed that the cells of some specimens can present two current morphologies for the pair 5, *i.e*., SM-SM and SM-A. The occurrence of structural heteromorphisms, like pericentric inversions and fissions, is known in crickets and they have been already demonstrated in Nemobiinae. Nonetheless, it is uncertain that a case of structural heteromorphism, such as a pericentric inversion, could have taken place in *Z. atlantica.* If so, intra-individual variation also would occur and three different karyotypes should be present.

Assuming that homozygosis occurs in submetacentric chromosomes 5, one could infer a case of mosaicism but it would be unlike that seen with polymorphic chromosomes. Even so, in *Z. atlantica* only two conditions for pair 5 were seen from cell to cell, *i.e*., one of the supposed homozygosis and other of heterozygosis, and it frequently appeared in the population as intra-individual variation. For this reason it has not been possible to obtain a quantitative evaluation of this character.

In the light of such evidence it is likely that the heteromorphism of pair 5 in *Z. atlantica* should be related to another kind of variation. Such variation probably means a differential activity of the genome from cell to cell under some specific conditions, or differential chromatin quantity in the genome. However, conventional staining analysis has not revealed its origin and these data remain opened to future research. Differential cytogenetic analysis could help to clear up these questions and add some new information on the karyotypical evolution of the group.
